# Oligo(dT) Fluorescence In Situ Hybridization to Visualize the Poly(A) mRNAs in the Internal Tissues of *Drosophila*

**DOI:** 10.21769/BioProtoc.5702

**Published:** 2026-06-05

**Authors:** Ankur Kumar, Jukta Biswas, Anand K. Singh

**Affiliations:** Department of Biology, Indian Institute of Science Education and Research Tirupati, Yerpedu, Tirupati, Andhra Pradesh, India

**Keywords:** Probe, FISH, Cytology, RNA, Drosophila, Microscopy

## Abstract

Fluorescence in situ hybridization (FISH) is a cytological method used to visualize specific oligonucleotide sequences within the cell. This method relies on the specific binding of a fluorescence-tagged probe, a short stretch of single-stranded polynucleotide, to its complementary sequence in the DNA or RNA, forming stable double-stranded hybrids. Fluorochromes, such as fluorescein, Alexa Fluor, cyanine dyes, or rhodamine, are attached to these probes to help in detecting their presence within the cell. Based on sequence complementarity, FISH allows for the visualization of the DNA or RNA with which they have hybridized. The distribution of these fluorochrome-tagged probes can be observed under a fluorescence or confocal microscope. The oligo(dT) FISH technique specifically utilizes a fluorochrome-tagged stretch of 40–50 thymidine (T) oligonucleotides that binds to the poly(A) tails of mature mRNAs within the cell. Newly transcribing pre-mRNAs and certain non-coding RNAs may not have poly(A) tails and therefore cannot be detected by this method. This step-by-step protocol outlines the oligo(dT) FISH technique for visualizing the cellular distribution of polyadenylated mRNAs in the tissues of *Drosophila* and other related model organisms.

Key features

• Oligo (dT) fluorescence in situ hybridization is a robust cytological technique to visualize polyadenylated RNAs in the cell.

• This oligo(dT) FISH protocol is applicable for a wide range of animals and cell culture model systems.

• This protocol is particularly useful to visualize mRNAs in thin tissues with a few layers of cells.

## Graphical overview


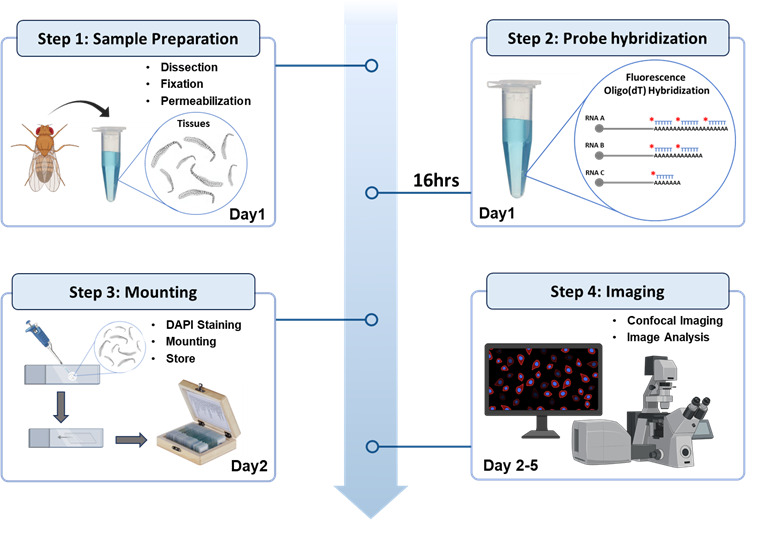


## Background

Fluorescence in situ hybridization (FISH) is a powerful technique for detecting specific nucleic acid sequences within fixed cells and tissues [1-3]. This technique has been widely used to visualize specific sequences in DNA (DNA-FISH) or RNA (RNA-FISH) molecules within the cell [4–7]. Traditional RNA-FISH uses gene-specific probes to localize individual transcripts. In contrast, the oligo(dT) FISH strategy employs a fluorescently labeled stretch of 40–50 thymidine (T) nucleotides that specifically hybridizes to the poly(A) tails of mature mRNAs [8,9]. Unlike gene-specific RNA in situ hybridization, which targets a specific transcript [10], this method allows for the visualization of the entire pool of poly(A) mRNAs [9]. This makes oligo(dT) FISH an essential tool for studying mRNA abundance and subcellular localization on a global scale. While earlier studies in yeast and mammalian systems have successfully utilized oligo(dT) probes to examine nuclear-cytoplasmic mRNA distribution, mRNA export defects, and stress-induced RNA retention, there is a notable absence of comprehensive, standardized oligo(dT) FISH protocols specifically optimized for *Drosophila* tissues [11–14]. This protocol can be widely applied to investigate mRNA dynamics during development, disease, and stress responses in *Drosophila, C. elegans*, or mammalian cell cultures. Furthermore, its compatibility with co-immunostaining makes it a versatile dual-track system for observing how specific proteins interact with the global mRNA pool under various cellular conditions.

## Materials and reagents

**Biological materials**

Set the desired cross in vials containing standard cornmeal food and collect the specific stage of *Drosophila* by anesthetizing them using ether in an etherizer. In this study, we used actively wandering third-instar larvae of *Nup107GFP/Nup107GFP* (Bloomington Stock No. 35514) genotype, which enables visualization of the nuclear membrane in the larval salivary gland.

**Reagents**

1. Ether (SRL, catalog number: 25049)

2. NaCl (Himedia, catalog number: MB023)

3. KCl (Himedia, catalog number: TC010)

4. Na_2_HPO_4_ (Himedia, catalog number: GRM168)

5. KH_2_PO_4_ (Himedia, catalog number: GRM1188)

6. Sodium citrate (Himedia, catalog number: TC526U)

7. NP-40 (Thermo, catalog number: 85124)

8. Triton X-100 (Himedia, catalog number: MB031)

9. 4′,6-diamidino-2-phenylindole (DAPI) (Thermo, catalog number: 62247)

10. Paraformaldehyde (PFA) (Himedia, catalog number: RM3660)

*Note: PFA is a polymer with a wide range of monomeric and oligomeric units of formaldehyde. It dissolves in water and depolymerizes into a monomeric form of formaldehyde. Formaldehyde can crosslink cellular constituents, particularly proteins with lysine residues. Formaldehyde is a toxic chemical; therefore, always wear a mask and gloves during handling.*

11. Diethyl pyrocarbonate (DEPC) (Himedia, catalog number: MB076)

*Note: DEPC irreversibly inactivates RNase contamination that may be present in dH_2_O. It is carcinogenic and, therefore, needs to be handled carefully.*

12. Yeast tRNA (Thermo, catalog number: AM7119)

*Note: Yeast tRNA is used as a nonspecific blocking agent, protecting both the probe and cellular RNA.*

13. Dextran sulfate (Merck, catalog number: 265152-M)

*Note: Dextran sulfate is a highly hydrated polysaccharide that acts as an effective volume exclusion agent. It affects the probe concentration by preventing its solubilization in water and thus gives higher hybridization rates in aqueous solutions.*

14. RNase inhibitor (Thermo, catalog number: EO0382)

*Note: RNase inhibitors directly bind with RNases in a non-competitive mode and thus block their activity. They have been widely used to stop the activity of RNases that are present within the cell. The presence of 1 U/μL RNase inhibitor can block most of the RNase activity during FISH.*

15. Formamide (SIGMA, catalog number: 15706)

*Note: Formamide interferes with hydrogen bonding in double-stranded polynucleotides and thus destabilizes double-strand formation. The presence of formamide in hybridization buffer, therefore, reduces the melting and annealing temperature, which keeps the tissue morphology relatively intact due to low incubation temperature. The working concentration of formamide in hybridization buffer varies from 10% to 50%, depending upon the probe length and its GC content.*

16. 4′,6-diamidino-2-phenylindole (DAPI) (Thermo, catalog number: 62247)

*Note: DAPI is a fluorescent stain that binds to the AT-rich region of DNA, with excitation at 405 nm and emission spectrum between 420 and 480 nm (blue color). The fluorescence of DAPI is enhanced several times following its binding with DNA.*

17. ProLong^TM^ Gold antifade mountant (Thermo, catalog number: P10114)

*Note: ProLong^TM^ Gold antifade mountant is a commercially available glycerol-based mounting media supplemented with an antifading agent that protects the fluorescent signal intensity.*

18. Riboprobe; probes used here for FISH are single-stranded, non-self-complementary short stretches of polynucleotides (20–1500 nucleotide long DNA or RNA) that have a complementary sequence to the target RNA. In the present study, we used RNase Free HPLC Purified 45 nucleotide long stretch of oligo(dT) probe tagged with Cy3 at the 5′ end. We customized the probe and procured it from Integrated DNA Technologies (IDT)

**Solutions**

1. 10× PBS (see Recipes)

2. 20× SSC (see Recipes)

3. 4% PFA (see Recipes)

4. 50% dextran sulfate (see Recipes)

5. 0.1% DEPC (see Recipes)

6. 0.3% PBST (see Recipes)

7. 100 mg/mL yeast tRNA (see Recipes)

8. 1 μg/mL DAPI (see Recipes)

9. Permeabilization buffer (see Recipes)

10. Hybridization buffer (see Recipes)

11. Wash buffer (see Recipes)

**Recipes**

**1. 10× PBS (pH 7.4)**

**Table:** 

Reagent	Concentration	Quantity
NaCl	1.37 M	80 g
KCl	27 mM	2 g
Na_2_HPO_4 _	100 mM	14.4 g
KH_2_PO_4 _	18 mM	2.45 g

Take 800 mL of distilled water (dH_2_O) and dissolve all four components. Adjust the pH to 7.4 and make up the volume to 1 L with dH_2_O. Autoclave and store the 10× PBS at room temperature (RT). Dilute the 10× PBS with dH_2_O in a 1:9 ratio to make the 1× PBS as a working solution and store it at 4 °C.

**2. 20× SSC (pH 7.0)**

**Table:** 

Reagent	Concentration	Quantity
NaCl	3 M	175.3 g
Sodium citrate	0.3 M	77.4 g

Take 800 mL of dH_2_O and dissolve both components. Adjust the pH to 7.0 with HCl and make up the volume to 1 L with dH_2_O. Autoclave and store at RT.

**3. 4% PFA**

Dissolve 4.0 g of PFA in 75 mL of 1× PBS at 60 °C with constant stirring for 10 min. Slowly add 5 N NaOH to the cloudy mixture until a clear solution is formed. Filter the solution with a Whatman filter paper and adjust the pH to 7.0 with HCl or NaOH. Make up the volume to 100 mL with 1× PBS and store in small aliquots at -20 °C. Defrost an aliquot and use it on the same day of the experiment. It is, however, preferred that small volumes of PFA solution be prepared fresh, as required. Prolonged storage of formaldehyde leads to its polymerization in the form of paraformaldehyde, thus reducing its fixative properties.

**4. 50% dextran sulfate**

Slowly dissolve 20 g of dextran sulfate powder in 30 mL of DEPC-treated dH_2_O and leave the tube overnight on the rotator. Make up the volume to 40 mL with dH_2_O and store at -20 °C in small aliquots. 50% dextran sulfate is very viscous, and special care should be taken during pipetting.

**5. 0.1% DEPC**

Mix 0.1 mL of DEPC in 100 mL of dH_2_O (0.1% DEPC) and autoclave after 4 h incubation at 37 °C. DEPC treatment is a very effective way to make RNase-free solutions; however, buffers like Tris or HEPES cannot be treated with DEPC as they absorb DEPC and make it unavailable for RNase. To prepare these buffers, DEPC-treated dH_2_O should be used to dissolve the RNase-free reagent powders.

**6. 0.3% PBST**

Triton X-100 is a non-ionic mild detergent that solubilizes proteins and is commonly used to permeabilize the cell membrane. Dissolve 0.03 mL of Triton X-100 in 10 mL of 1× PBS to make 0.3% PBST, which is used as a washing buffer in FISH.

**7. 100 mg/mL yeast tRNA**

Dissolve 25 mg of yeast tRNA powder in 250 μL of DEPC-treated dH_2_O and store at -20 °C.

**8. 1 μg/mL DAPI**

Dissolve 1 mg of DAPI in 1 mL of dH_2_O and store the stock (1 mg/mL) solution at -20 °C. Dilute the DAPI stock solution in 1× PBS (1:1000) to make the 1 μg/mL working DAPI solution. Store at 4 °C for several weeks.

**9. Permeabilization buffer**

**Table:** 

Reagent	Concentration	Quantity
Triton X-100	1%	1 μL
NP-40	1%	1 μL
40 U/μL RNase inhibitor	1 U/μL	2.5 μL
1× PBS		95.5 μL

Dissolve 1 μL of Triton X-100, 1 μL of NP-40, and 2.5 μL of 40 U/μL RNase-inhibitor in 95.5 μL of 1× PBS. Use fresh.

**10. Hybridization buffer**

**Table:** 

Reagent	Concentration	Quantity
Formamide	25%	25 μL
20× SSC	2×	10 μL
50% dextran sulfate	10%	20 μL
100 mg/mL yeast tRNA	1 mg/mL	1 μL
100 ng/mL Oligo(dT) riboprobe	5 ng/mL	5 μL
DEPC-treated dH_2_O		39 μL

Take 39 μL of DEPC-treated dH_2_O to dissolve 25 μL of formamide, 10 μL of 20× SSC pH 7.0, 20 μL of 50% dextran sulfate, 1 μL of 100 mg/mL yeast tRNA, and 5 μL of 100 ng/μL Cy3-labeled oligo(dT) riboprobe. Use fresh.

**11. Wash buffer**

**Table:** 

Reagent	Concentration	Quantity
Formamide	25%	25 μL
20× SSC	2×	10 μL
DEPC-treated dH_2_O		65 μL

Dissolve 25 μL of formamide and 10 μL of 20× SSC in 65 μL of DEPC-treated dH_2_O. Use fresh.

**Laboratory supplies**

1. Glass slide (Blue Star microslide, 75 mm × 25 mm)

2. Fine forceps (Dumont Tweezer, catalog number: 72701-D)

3. Glass coverslips (Blue Star, 22 mm2, part no. CSI-130)

4. Transparent Nail polish (Shade-40, Lakme, Model No TWUY100)

5. Dissection needle (Syringes 31G, Dispovan, RDMS-01)

6. Round synthetic paintbrush (Round paintbrush Size 4)

7. Dark chamber (opaque plastic box)

8. Etherizer (Tarson, catalog numbers: 630020 and 500043)

## Equipment

1. Stereomicroscope (Nikon, model: SMZ745)

2. Fluorescence microscope (Olympus, model: IX83)

3. Confocal microscope (Olympus, model: FV3000)

4. Incubators set at 25 °C and 42 °C (Orbitek, model: CINC450)

## Software and datasets

1. Fiji/ImageJ software (NIH, USA) (Java 6, available at imagej.nih.gov/ij)

## Procedure

**A. Tissue pretreatment**

1. Collect actively wandering, healthy third-instar larvae in a Petri dish.

2. Wash larvae with distilled water (dH_2_O) and dry them on tissue paper.

3. Dissect out the desired internal tissues of larvae in 1× PBS on a glass slide with the help of fine forceps or dissecting needles.

4. Fix the tissues by incubating them in freshly prepared 4% PFA at RT for 20 min in a microcentrifuge tube. Perform all FISH steps in the same microcentrifuge tube.

*Note: Fix the tissue immediately after dissection to minimize any stress to the tissue. Ensure that the tissues are properly submerged in the fixing solution and not adhering to the container wall or floating on the surface.*

5. Remove the fixative and wash the tissues with 200 μL of 0.3% PBST on a rotator for 10 min at RT. Repeat this washing step three times.

6. Incubate the tissue with permeabilization buffer for 20 min at 4 °C on a rotator to permeabilize the cell and nuclear membrane.

7. Rinse the tissue with 200 μL of 0.3% PBST three times for 10 min each.

8. Rinse the tissue further with 200 μL of 2× SSC twice for 10 min each.

**B. Probe hybridization**

1. For each sample of 5–6 larval tissue, prepare ~30 μL of hybridization solution. Thoroughly mix all components with a 200 μL pipette.

2. Incubate the tissue with hybridization solution in a 500 μL microcentrifuge tube at 42 °C for 12–16 h. Make sure that the cap of the tube is properly closed and sealed with parafilm during incubation to prevent drying.

*Note: The composition of the hybridization buffer and incubation temperature may vary with probe length and its GC content. Smaller probes (~30 nt) need a relatively lower concentration of formamide (10%) and lower incubation temperature (35–37 °C), while longer probes (~500 nt) need a higher concentration of formamide (50%) and higher incubation temperature (60–65 °C).*

3. Rinse the tissue at 42 °C with freshly prepared prewarmed wash buffer three times for 5 min each.

4. Rinse the tissue again at 42 °C with prewarmed 2× SSC, followed by 1× SSC, three times for 5 min each.

5. Rinse the tissue further with 1× PBS at RT three times for 5 min each.

*Note: Immunostaining for a protein of interest can be performed to examine its interaction with poly(A) RNA in the cell. Immunostaining can be performed just after completion of FISH and before counterstaining with DAPI [2,7,15,16].*

**C. Counterstaining and microscopy**

1. Counterstain the nuclei with DAPI (1 μg/mL) by incubating the tissue for 10 min at RT. Mount the tissue in antifade mounting media. Store the slides in a dark place at -20 °C until further examination.

2. Examine the fluorescence signals using appropriate excitation and emission filters under a fluorescence or confocal microscope.

*Note: The excitation and emission spectra of the fluorochromes used in FISH should be sufficiently different for clear distinction of the two signals (without any* bleeding *effect).*

## Data analysis

**Figure 1.**
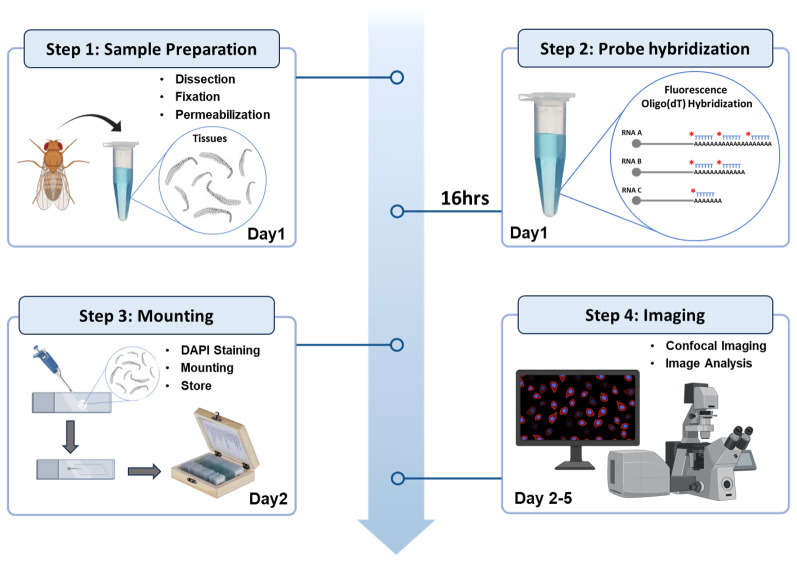
Oligo(dT) fluorescence in situ hybridization. (A–C) Confocal single-section image of Cy3-labeled oligo(dT) FISH showing the distribution of polyadenylated mRNAs (red) in the salivary gland of third-instar larva. The nuclear membrane is visualized by the GFP-tagged Nup107 protein (green). Nuclei are counterstained with DAPI (blue). Scale bars = 20 μm. (D) Oligo(dT) probe fluorescence intensity in the nucleus and cytoplasm of the salivary gland (n = 50). The difference in signal intensity was statistically significant (**** represents p < 0.001) tested by the unpaired t-test.

In the example shown in Figure 1, we conducted RNA FISH with a Cy3-labeled oligo(dT) riboprobe in the salivary glands of a third-instar larva. The Nup107GFP protein is visualized at the nuclear membrane, clearly outlining the boundary between the nucleus and the cytoplasm. The image indicates that poly(A) mRNA is present in both the nucleus and the cytoplasm (Figure 1A–C). We measured fluorescence signal intensity using the *Analyse* > *Measure* tool in FIJI and found that the level of poly(A) mRNA in the cytoplasm was significantly higher than that in the nucleus (Figure 1D). Notably, the oligo(dT) FISH signal was nearly absent in the nucleolus, which is primarily composed of the non-polyadenylated ribosomal RNAs (rRNAs) and small nucleolar RNAs (snoRNAs).

## Validation of protocol

This protocol has been used and validated in the following research article:

- Singh et al. [9]. The RNA helicase UPF1 associates with mRNAs co-transcriptionally and is required for the release of mRNAs from gene loci. *eLife.*

## General notes and troubleshooting

**General notes**

1. Experimental controls for FISH: In order to ensure that the observed FISH signal is genuine and not a false-positive result, positive and negative experimental controls should also be processed in parallel. The positive control could be a widely expressed and well-studied gene with a known expression pattern, like tubulin or G3PDH. FISH with a probe against an mRNA that is expressed only in some specific cell types can also be used as a good control to confirm the specificity of the signal. Likewise, a negative control could be probed with the sense strand sequence of the same gene, which is expected to have no complementary sequence and, therefore, should not generate any hybridization signal. However, many genes express the antisense strand as well and, therefore, can also give a positive signal with the sense probe. Another negative control can be a parallel sample treated with RNase, which must not produce any FISH signal. However, great care is needed, as even a very small RNase contamination of the experimental sample can ruin the entire experiment.

2. RNases are the natural enemy of RNA molecules, and blocking their activity is a big challenge in FISH. They can be present anywhere, including reagents, solutions, instruments, and, most importantly, on our body surface. Therefore, special care is required in all experiments involving RNA. Some RNases are resistant to standard decontamination methods. Therefore, all the reagents should be kept in an RNase-free environment. The solutions and dH_2_O used in the experiment should be treated with DEPC. The working bench, pipettes, and plasticware should be wiped with 0.2 N NaOH and then with hot dH_2_O. All glassware should be treated with 0.5% SDS and baked (dry heating at 150 °C) after washing. The tubes and tips should be fresh and stored in properly sealed containers. It is important to wear disposable plastic or latex gloves to keep the sample safe from RNases present on our hands. If the glove has come into contact with RNase-contaminated lab equipment, such as pipettes, microscopes, or door handles, it should be immediately changed, as it would no longer be RNase-free.

3. Keep the probe stock at -80 °C and an aliquot of working concentration at -20 °C, as multiple freeze/thaw cycles can degrade the probe. The box containing the probe should be opaque to ensure a minimal possibility of photobleaching.

4. A few tissues, like the *Drosophila* embryo, have a thick cuticle layer. In order to perform FISH in an embryo, we need to perform dechorionation to ensure the proper penetration of the probe inside the embryo. Incubating the embryo with a 3% bleach solution for 90 s offers efficient dechorionation [17]. Likewise, tissues like adult brain need a higher concentration of detergent in washing buffer (0.5% PBST) to penetrate the probe. Tissues like Malpighian tubule, gut, fat body, etc. need an even smaller concentration of washing buffer (0.1% PBST).

5. Stopping the experiment at any step of the protocol can cause variability in the results. Therefore, it is suggested to complete the FISH protocol without any pause.

**Troubleshooting**

1. The sequence of the probe should be very specific and complementary to the RNA one wants to localize. The concentration of probe in hybridization buffer must be in an adequate range, as a very low or high concentration may lead either to no signal or to a nonspecific signal, respectively.

2. The concentration of Triton X-100 in the rinsing solution varies with tissue type, e.g., tissues with a thick outer membrane/sheath (larval salivary gland and brain) need a higher concentration (0.5%), while tissues with a thin outer membrane (larval imaginal disc, Malpighian tubules, fat body, etc.) need a low (0.1%) concentration of the detergent.
